# Global research trends in extracellular vesicles based on stem cells from 1991 to 2021: A bibliometric and visualized study

**DOI:** 10.3389/fbioe.2022.956058

**Published:** 2022-08-30

**Authors:** Jianjing Lin, Zhen Yang, Li Wang, Dan Xing, Jianhao Lin

**Affiliations:** ^1^ Arthritis Clinical and Research Center, Peking University People’s Hospital, Beijing, China; ^2^ Arthritis Institute, Peking University, Beijing, China; ^3^ Department of Sports Medicine and Rehabilitation, Peking University Shenzhen Hospital, Shenzhen, China; ^4^ Department of Biomedical Engineering, Institute of Future Technology, Peking University, Beijing, China

**Keywords:** global research trend, stem cells, extracellular vesicles, bibliometrics, visualized study

## Abstract

**Objective:** With the development of extracellular vesicles (EVs) based on stem cells research all over the world, our present study was aiming to discover the global trends in this field.

**Methods:** All publications related to EVs based on stem cells from 1991 to 2021 were collected from the Science Citation Index-Expanded of Web of Science Subsequently, the data were evaluated using the bibliometric methodology. In terms of visualized study, the VOS viewer software was performed to investigate the bibliographic coupling, co-citation, co-authorship, and co-occurrence trends, and last for the publication’s trends involved in the field of EVs based on stem cells.

**Results:** A total of 8,208 publications were retrieved and the relative number of global publications and research interests were increasing every year especially in recent 5 years. China rank top one in terms of total publications, prolific authors, and funds, whereas the USA made the greatest contributions with the most total citations and highest H-index to the global research. Stem cell research therapy contributed the highest publications, whereas the journal of PLOS ONE showed the best total link strength. The Shanghai Jiao Tong University, University of California System, and Harvard University were the most contributive institutions. The global studies could be divided into six clusters as follows: cancer research, musculoskeletal system research, respiratory system research, urinary system and endocrine system research, nerve system research, and cardiovascular system research. All the directions were predicted to still hotspots in near future researches in this field.

**Conclusion:** The total number of publications about EVs based stem cells would be increasing according to the current global trends. China and the USA was the largest contributors in this field. Further efforts should be put in the directions of cancer research, musculoskeletal system research, respiratory system research, urinary system and endocrine system research, nerve system research, as well was cardiovascular system research in this field of EVs based stem cells.

## Introduction

Stem cells are defined as undifferentiated cells by the ability to self-renew, differentiate into different types of cells, proliferate and regenerate tissues. Stem cells have been widely used in preclinical and clinical studies of regenerative medicine, such as osteoarthritis (OA) (Xing et al., 2020a; Xing et al., 2020b), cartilage defect (Ning et al., 2019; Li et al., 2021), diabetes (Zhang et al., 2021), heart injury (Tang et al., 2018) and so on. However, there is still a huge challenge in the widespread application of stem cells in regenerative therapy. The clinical use of embryonic stem cells (ESCs) is limited by the ethical considerations and legal restrictions (de Miguel-Beriain, 2015). There is a risk of tumor formation after injection induced pluripotent stem cells (iPSCs) into the host (Blin et al., 2010). There are also some drawbacks in mesenchymal stem cells (MSCs), such as low survival rate *in vivo* and potential immune rejection (Rong et al., 2021). At present, more and more studies have showed that stem cells are considered to exhibit their therapeutic effect in a paracrine manner and extracellular vesicles (EVs) play a pivotal role in this manner (Liang et al., 2014; Maacha et al., 2020).

Extracellular vesicles (EVs), as a lipid bilayer membrane structure released from cells, cannot replicate and have no functional nucleus, covering exosomes, microvesicles, microparticles, exosomes, tumors, apoptotic bodies and many other names (Théry et al., 2018). EVs are considered as a promising alternative strategy for stem cells therapy with many potential advantages, such as higher stability, lower manufacturing costs, more convenient sterilization and storage (Liao et al., 2021a). At present, EVs have been more and more applicated in OA (Rong et al., 2021), intervertebral disc degeneration (Liao et al., 2021b), myocardial infarction (Liu et al., 2020) and other studies. However, studies on quantitative and qualitative characteristics of global research on EVs based on stem cells are still limited. It is necessary to evaluate the current status and trends of EVs and stem cells research which can predict promising hot topics and directions in this field.

As the central part of scientific research, publications are treated as an important indicator of the research trends and contributions. Based on the literature metrology characteristics and literature databases, bibliometric analysis can provide qualitative and quantitative information to assess the trends of research activities over time. Bibliometric analysis can predict the development in a certain field and by comparing the contributions of authors, journals, institutes, countries and regions (Pu et al., 2016). Furthermore, it is also used in the formulation of clinical policy and the provision of guidelines (Avcu et al., 2015). At present, this viable methods has been successfully applied in evaluating research trends in OA (Xing et al., 2018; Wang et al., 2019a), exosome (Wang et al., 2019b), dental stem cells (Zhao et al., 2021), haze (Li et al., 2017), microbiome (Zyoud et al., 2019) and many other studies. However, the global development trends of EVs based on stem cells have not been well studied yet. The purpose of our study was to evaluate the current status and global trends of EVs research in stem cells.

## Materials and methods

### Data source

All publications originating from Web of Science (WoS), including Science Citation Index Expanded (SCIE), Social Sciences Citation Index, Arts & Humanities Citation Index, Conference Proceedings Citation Index-Science, Conference Proceedings Citation Index-Social Science and humanities, Emerging Sources Citation Index, Current Chemical Reactions and Index Chemicus, which contains more than 12,000 international academic journals, is one of the most comprehensive and authoritative database platforms to obtain global academic information (Wu et al., 2021a), were analyzed using bibliometric analysis according to previous studies (Liao et al., 2021b; Wu et al., 2021b; Liu et al., 2021; Yan et al., 2021).

### Search strategy

All the published papers were collected from WoS and the database expiration date was set to 9 February 2022. In this study, the search terms were shown as follows: theme = stem cells*AND theme = Extracellular Vesicles or exosomes or microvesicles or microparticles or ectosomes or oncosomes or apoptotic bodies AND Language = (English) AND Document types = (ARTICLE OR REVIEW). Additionally, the detailed information of certain countries of regions was refined by indexing country/region in the WoS. The publication criteria were shown as follows: 1) The manuscript focused on the theme of EVs based on stem cells; 2) The document types are Article and Review. 3) The papers must be written in English. The exclusion criteria were also shown as follows: 1) The themes were not related to EVs based on stem cells; 2) Articles were briefings, news, meeting abstracts, etc.

### Data collection

All the records of publications, including year of publication, title, authors’ names, affiliations, nationalities, abstract, keywords, and name of publishing journals were saved as. txt files from WoS database and then imported into Excel 2021. Coauthors (LJJ and YZ) independently browsed and extracted all these data from these publications. Any disagreement was resolved by discussion with experts to reach a final consensus. Finally, all the authors separately cleaned and analyzed the data with GraphPadPrism 8.

### Bibliometric analysis

As we aforementioned, the intrinsic function of WoS was to characterize the basic features of eligible publications. Total global publications of each year and the relative research interest (RRI) was defined as the number of publications in one certain field by all field publications per year which was searched from WoS and the publishing year was set as (1991-01-01 to 2021-12-31) (Xing et al., 2018; Wang et al., 2019b) were analyzed by GraphPadPrism 8. The world map was performed by R software including python + numpy + scipy + matplotlib and the time curve of publications was depicted as: f (x) = c/(1+a × exp [-b × (x-1994)]) according to previous article (Xing et al., 2018), whereas the difference in our paper was that the year 1994 replaced by 1991 based on the first published date**.** The specific code are shown as follows:

Import numpy as np

From scipy. optimize import curve_fit

Def logistic_growth_function (t, K, P0):

T0 = 1994

R = 0.65

Exp_v = np. exp (r *(t - t0))

Return (K * exp_v *P0)/(K + (exp_v -1) *P0).

Popt, pcov = curve_fit (logistic_growth_function, x, y).

Y_t = logistic_growth_function (x_t, popt [0], popt [1])

In the formula, the independent variable x indicates to the publishing year and the f(x) means the cumulative amount of publications. Additionally, formula T = lna/b + 1994 was performed to calculate the inflection point indicating the time that the growth rate of publications changes from positive to negative. The data of total publications from top 25 countries were acquired from WoS and analyzed by GraphPadPrism 8. Additionally, the total citation, citation frequency and H-index level were analyzed by GraphPadPrism 8. The H-index, indicating that a scholar has published H papers and they have been cited at least H times, was created to measure the impact of scientific research. Hence, it reflects both the number of publications and corresponding citations (Bertoli-Barsotti and Lando, 2017). In addition, high-contribution journals, authors, research orientations, institutions and funds of global publications were also analyzed by GraphPadPrism 8.

### Visualized analysis

The VOS viewer (Leiden University, Leiden, Netherlands) was a powerful software for constructing and visualizing bibliometric networks and thus was chosen for the visualization of the publications in our present study. Specially, the VOS viewer was performed for bibliographic coupling, co-citation, co-authorship and co-occurrence analyses**.** We used CiteSpace to analyze the cluster of keywords with strong citations bursts. The parameters of CiteSpace were set as follows: link retaining factor (LRF = 3), look back years (LBY = 5), e for top N (e = 1), time span (1991–2021), years per slice (1), links (strength: cosine, scope: within slices), selection criteria (g-index: k = 25), and minimum duration (MD = 2 for keywords; MD = 5 for references).

## Results

### Trend of global publications

**FIGURE 1:**
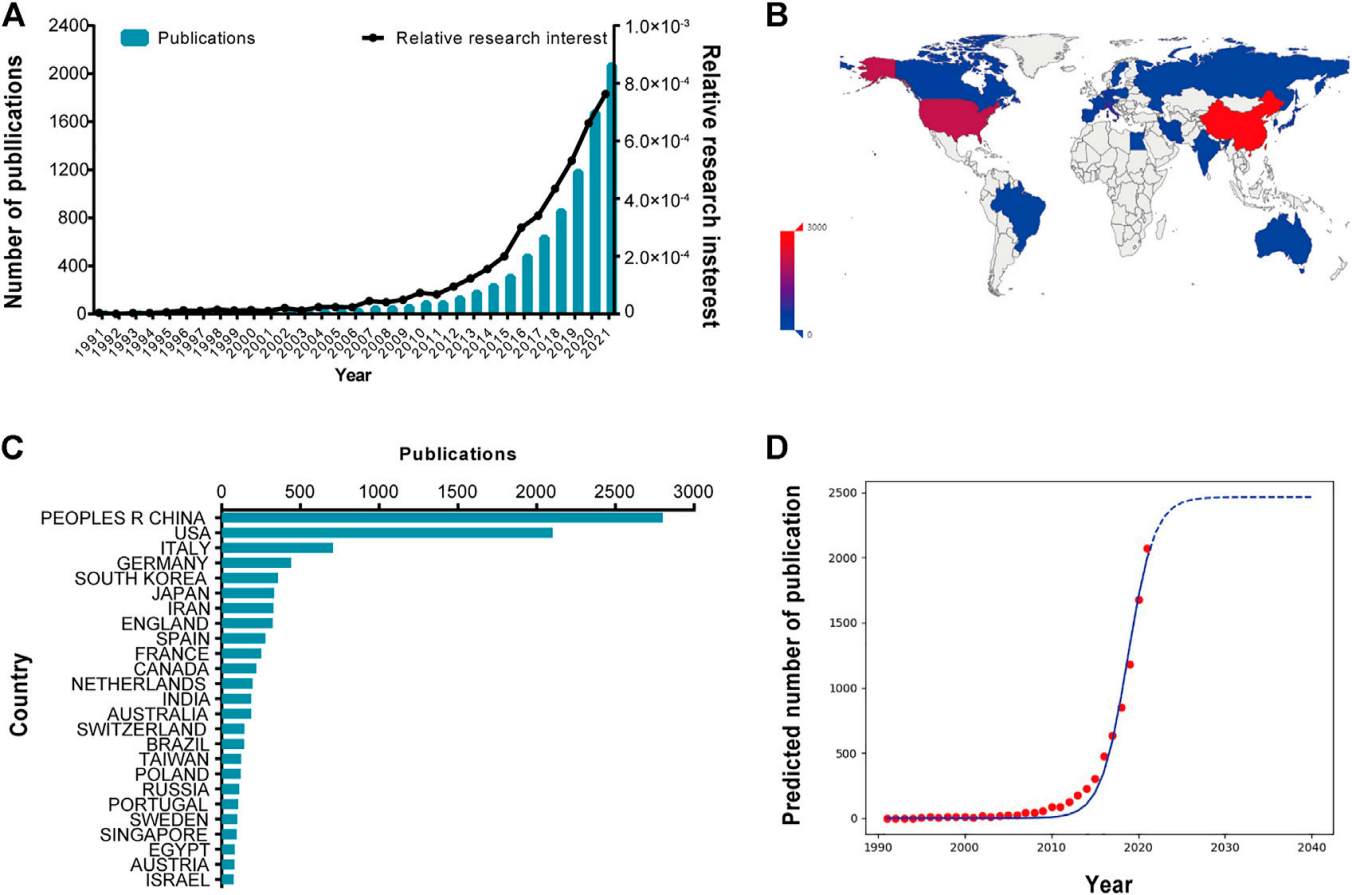
Global trends and countries/regions contributing to extracellular vesicles (EVs) based on stem cells research. **(A)** The global number and relative research interests of publications related to EVs based on stem cells research. The blue bars mean the publication numbers each year, and the black curve meas the relative research interests. **(B)** Distribution of EVs based on stem cells research in world map. **(C)** The sum of EVs based on stem cells related publications from the top 25 countries and regions. **(D)** Model fitting curves of global trends in publications of EVs based on stem cells.

In terms of global publications, a total of 8,208 publications met the search criteria. From 1991 to 2021, the trend of global publications increased steadily year by year. The amount of publications increased from 2 1991) to 2069 (2021). Most research was published in 2021 (2069, 25.2071%) (Figure 1A). As well as the contributions of countries and regions analysis, in total, 93 countries and regions made contributions in publications in this domain. China published the most papers (2,800, 34.113%), followed by the United States (2,101, 25.597%), Italy (707, 8.614%), Germany (441, 5.373%) and South Korea (358, 4.362%) (Figure 1B,C).In order to predict the future global publications trend, a logistic regression model was used to create a time curve of the number of publications. The model fitting curves of the growth trends was shown in Figure 1D. According to the time curve, the number of publications in this field was estimated to increase from two in 1991 to approximately 2,463 by 2040.

### Quality of publications of different countries and regions

**FIGURE 2:**
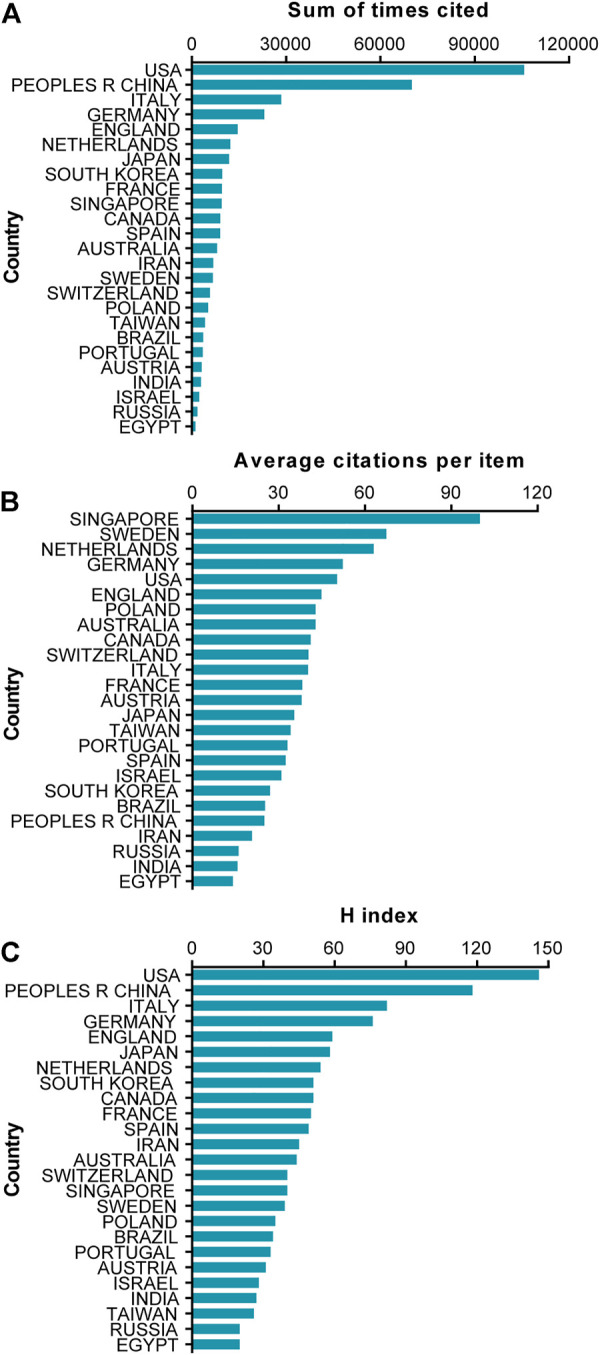
Citation frequency and H index levels of different countries and regions. **(A)** The top 25 countries and regions of total citations of EVs based on stem cells research publications. **(B)** The top 25 countries and regions of the average citations per paper of EVs based on stem cells research publications. **(C)** The top 25 countries and regions of the H-index of EVs based on stem cells research publications.

Regards to the total citation frequency analysis, publications from the United States had the highest total citation frequencies (105698). China ranked second in total citation frequencies (70010), followed by Italy (28462), Germany (23061) and England (14554) (Figure 2A). Furthermore, publications from Singapore had the highest average citation frequencies (99.97). Sweden ranked second in average citation frequency (67.45), followed by Netherlands (63.03), Germany (52.29) and the United States (50.31) (Figure 2B). Additionally, the relative publications from the United States had the highest H-index (146), followed by China (118), Italy (82), Germany (76) and England (59) (Figure 2C).

### Analysis of global leading journals, authors, research orientation, institution output, and funding sources

**FIGURE 3:**
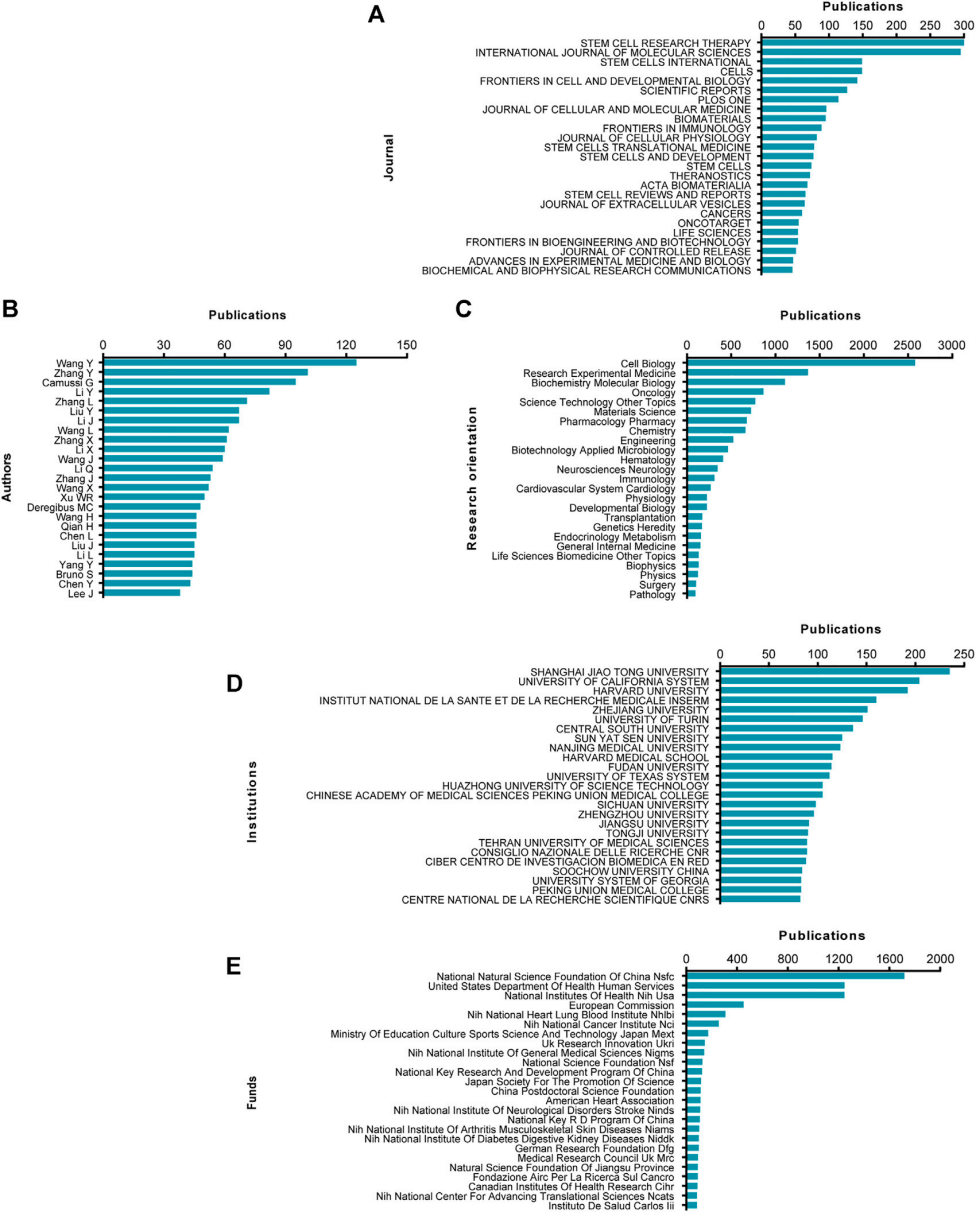
High-contribution journals, authors, research orientations, institutions and funds of global publications about EVs based on stem cells. **(A)** The top 25 journals with most publications related to EVs based on stem cells. **(B)** The top 25 authors with most publications related to EVs based on stem cells. **(C)** The top 25 research orientations with most publications related to EVs based on stem cells. **(D)** The top 25 institutions with most publications related to EVs based on stem cells. **(E)** The top 25 funds with most publications related to EVs and stem cells.

In terms of leading journals and cited journals analysis, *Stem Cell Research & Therapy* (impact factor [IF] = 6.832, 2020) published the most with 300 publications. There were 295 publications in *International Journal of Molecular Sciences* (IF = 5.924, 2020), 149 publications in *Stem Cells International* (IF = 5.443, 2020), 149 articles in *Cells* (IF = 6.6, 2020) and 142 publications in *Frontiers in Cell and Developmental Biology* (IF = 6.684, 2020). The top 25 journals with the most publications were listed in Figure 3A. For the leading authors and cited authors analysis, the top 25 authors contributed a total of 1504 publications, which accounted for 18.3236% of all publications in this field. Wang Y published the most research with 125 publications, followed by Zhang Y with 101 publications and Camussi G with 95 publications (Figure 3B).

Figure 3C showed the top 25 research orientations related to EVs based on stem cells. The most prevalent research fields were cell biology, research experimental medicine, biochemistry molecular biology, oncology and science technology other topics.

The top 25 contributive institutions were listed in Figure 3D. Shanghai Jiao Tong University published the most (235 publications), University of California System ranked second (204 publications), while Harvard University ranked third (192 publications).

The top 25 funding sources were shown in Figure 3E. In totally, 1718 publications were funded by National Natural Science Foundation of China (NSFC) (ranked first), 1247 publications were funded by United States Department of Health Human Services (ranked second) and 1246 publications were funded by National Institutes of Health (NIH) (ranked third).

### Bibliographic coupling analysis of institution, journal, country, and author

**FIGURE 4:**
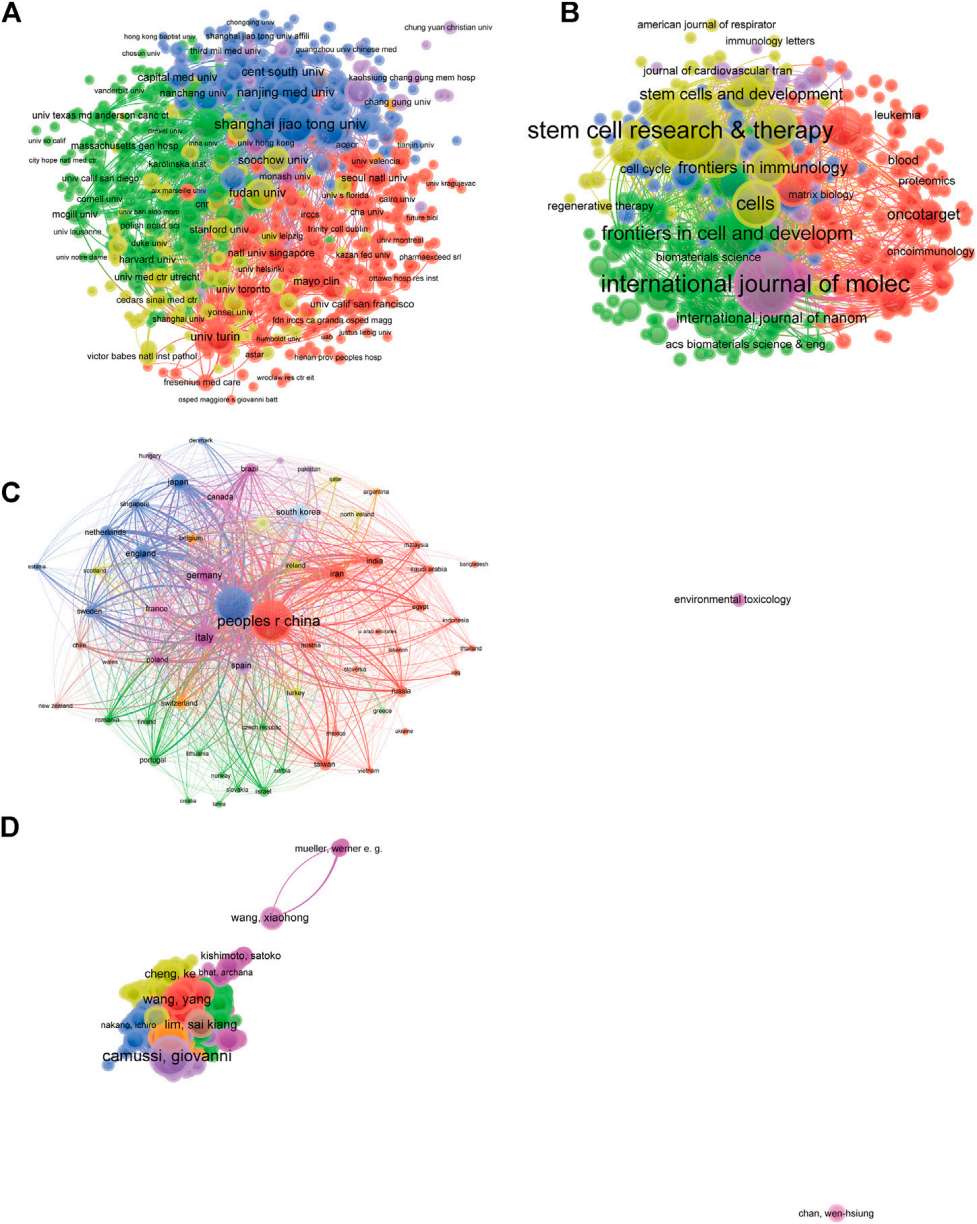
Mapping of bibliographic coupling analysis related to EVs based on stem cells. **(A)** Mapping of the 354 identified journals on EVs based on stem cells. **(B)** Mapping of the 834 institutions on EVs based on stem cells. **(C)** Mapping of the 62 countries on EVs based on stem cells. **(D)** Mapping of the 1,090 authors on EVs based on stem cells. The line between different points represents that the journals/institutions/countries/authors had establish a similarity relationship. The thicker the line, the closer the link between the journals/institutions/countries/authors.

Papers (defined as the minimum number of documents of an organization that were used more than five and the maximum number of organizations per document no more than 25) were identified in the 834 institutions and analyzed using VOS viewer (Figure 4A). The top five institutions with largest total link strength were shown as follows: Shanghai Jiao Tong University (total link strength = 965239 times), University of Turin (total link strength = 772674 times), Zhejiang University (total link strength = 750821 times), Harvard Medica l School (total link strength = 725276 times), and Central South University (total link strength = 545210 times).

The bibliographic coupling was used to analyze the similarity relationship between documents. Firstly, we used VOS viewer to analyze the name of journals in total publications. There are 354 identified journals appeared in total link strength which were shown in Figure 4B. The top five journals with largest total link strength were shown as follows: *International Journal of Molecular Sciences* (Impact Factor, IF = 5.924, 2020, total link strength = 877707 times), *Stem Cells Research & Therapy* (Impact Factor, IF = 6.832, 2020, total link strength = 625978 times), *Cells* (IF = 6.6, 2020, total link strength = 526806 times), *Stem Cells International* (Impact Factor, IF = 5.443, 2020, total link strength = 436220 times) and *Frontiers in Cell and Developmental Biology* (Impact Factor, IF = 6.684, 2020, total link strength = 415911 times).

Publications (defined as the minimum number of documents of a country more than 5) originating from 62 countries were analyzed via VOS viewer (Figure 4C). The top five countries with largest total link strength were as follows: China (total link strength = 7181891 times), USA (total link strength = 6679108 times), Italy (total link strength = 2921586 times), Germany (total link strength = 1620512 times) and Iran (total link strength = 1541782 times).

Publications (defined as the minimum number of documents of an author more than 5) were produced from 1,090 authors and were further analyzed by VOS viewer. As shown in Figure 4D, the top five productive authors are shown below: Camussi, Giovanni (total link strength = 658260 times), Bruno, Stefania (total link strength = 345306 times), Deregibus, Mari a chiara (total link strength = 334206 times), Lim, Sai kiang (total link strength = 261076 times), Xu, Wenrong (total link strength = 260656 times).

### Co-citation analysis of cited reference, journal and author

**FIGURE 5:**
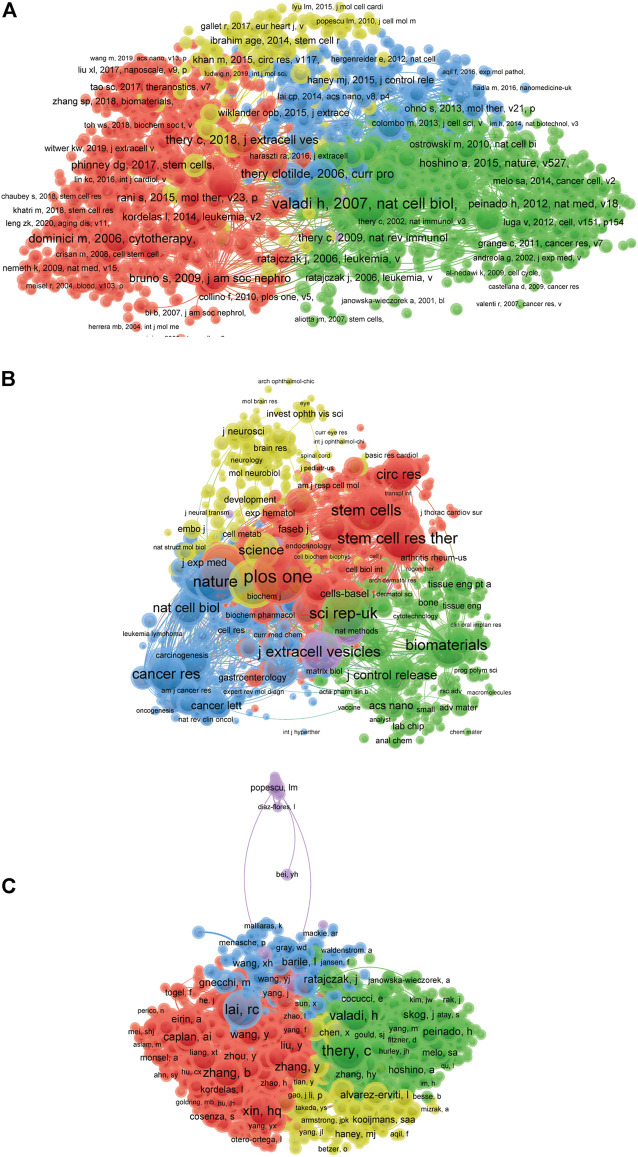
Mapping of co-citation related to EVs based on stem cells. **(A)** Mapping of the co-cited references related to this field (The 927 points with different colors represent the 927 cited references.) **(B)** Mapping of the co-cited journals related to this field (The 1,269 points with different colors represent the 1,269 identified journals.) **(C)** Mapping of the co-cited authors related to this field (The 1,558 points with different colors represent the 1,558 identified journals.) The point sizes represent the citation frequency. The line between different points indicates that they were cited in one paper. The shorter the line, the closer the link between two papers. The same color of the points represents the same research area they belong to.

The purpose of co-citation analysis is to reveal the relatedness of items based on the total citied number and there are 927 references were analyzed via VOS viewer (defined as the minimum number of documents of a cited reference more than 50) (Figure 5A). The top five articles with greatest total link strength were as follows: Valadi h, 2007, NAT CELL BIOL, v9 (total link strength = 33746 times); Lai rc, 2010, STEM CELL RES, v4 (total link strength = 22853 times); Raposo g, 2013, J CELL BIOL, v200 (total link strength = 21618 times); Alvarez-ervitil, 2011, NAT BIOTECHNOL, v29 (total link strength = 20596 times); Skog j, 2008, NAT CELL BIOL, v10 (total link strength = 17098 times).

The names of journals of co-citation analysis were performed using VOS viewer and the journal with minimum number of citations over 50 were defined. As illustrated in Figure 5B, 1,269 journals were shown in the total link strength. The top five journals with best total link strength were as follows: *Plos One* (total link strength = 1500546 times), *p Nani Acad Sci USA* (total link strength = 1222500 times), *Nature* (total link strength = 1074106 times), *Sci Rep-UK* (total link strength = 1073019 times), and *Stem Cells* (total link strength = 955338 times).

The co-citation analysis was to consider the relatedness of the items based on the numbers they were co-cited. Total of 1,558 authors with the minimum 50 documents were analyzed using VOS viewer (Figure 5C). The top five publications with largest total link strength were as follows: Thery, C (total link strength = 91041 times), Lai, Rc (total link strength = 69068 times), Raposo, G (total link strength = 59242 times), Xin, Hq (total link strength = 55895 times), and Valadi, H (total link strength = 54694 times).

### Co-authorship analysis of author, institution, and country

**FIGURE 6:**
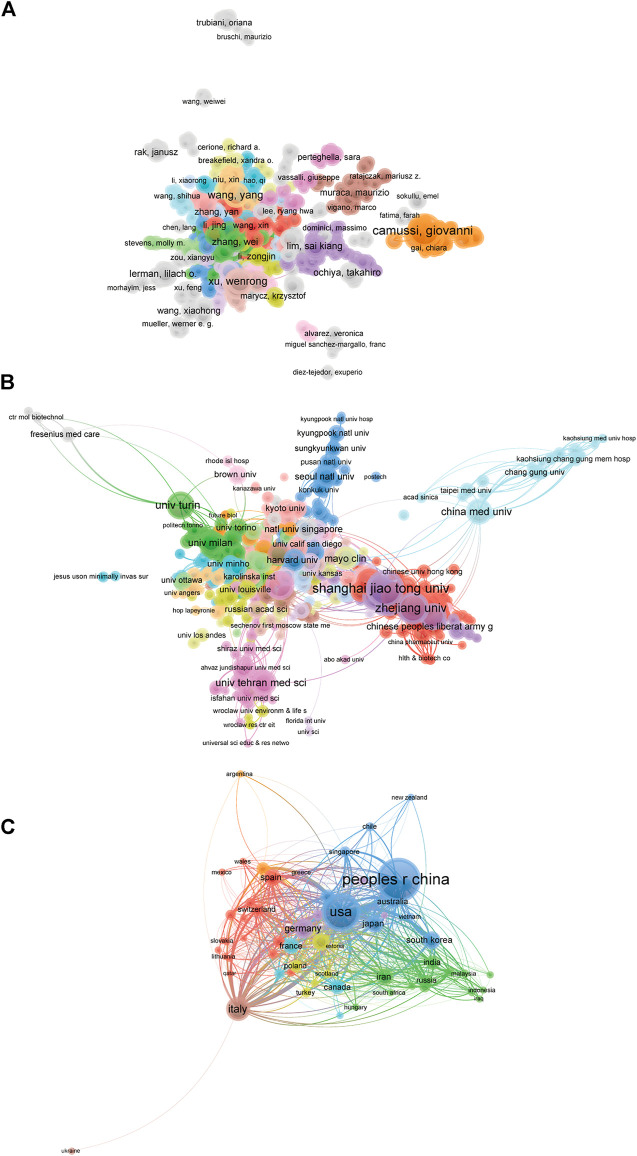
Co-authorship analysis of global research about EVs based on stem cells. **(A)** Mapping of the 1090-author co-authorship analysis on EVs based on stem cells. **(B)** Mapping of the 834-institution co-authorship analysis on EVs based on stem cells. **(C)** Mapping of the 62-country co-authorship analysis on EVs based on stem cells. The line between two points represents the establishment of collaboration within two authors/institutions/countries, where the thickness indicates the collaboration strength between the two authors/institutions/countries.

Co-authorship analysis was performed to evaluate the items relatedness based on the total number of coauthored papers. There are 1,090 authors with over five documents were analyzed using VOS viewer and the results were shown in Figure 6A. The top five authors with largest total link strength were as follows: Camussi, Giovanni (total link strength = 341 times), Xu, Wenrong (total link strength = 236 times), Qian, Hui (total link strength = 226 times), Bruno, Stefania (total link strength = 190 times), Deregibus, Maria chiara (total link strength = 175 times).

There are 834 institutions with more than five documents were analyzed through VOS viewer (Figure 6B). The top five institutions with greatest total link strength were shown below: Harvard Medical School (total link strength = 313 times), Shanghai Jiao Tong University (total link strength = 207 times), Tehran University Medical Sciences (total link strength = 182 times), Sun Yat Sen University (total link strength = 147 times), Massachusetts General Hospital (total link strength = 146 times).

There are 62 countries with over five papers were chosen and analyzed using VOS viewer and the results were depicted in Figure 6C. The top five countries with greatest total link strength were the following: USA (total link strength = 1,388 times), China (total link strength = 626 times), Italy (total link strength = 522 times), Germany (total link strength = 494 times) and England (total link strength = 406 times).

### Co-occurrence analysis of keywords

**FIGURE 7:**
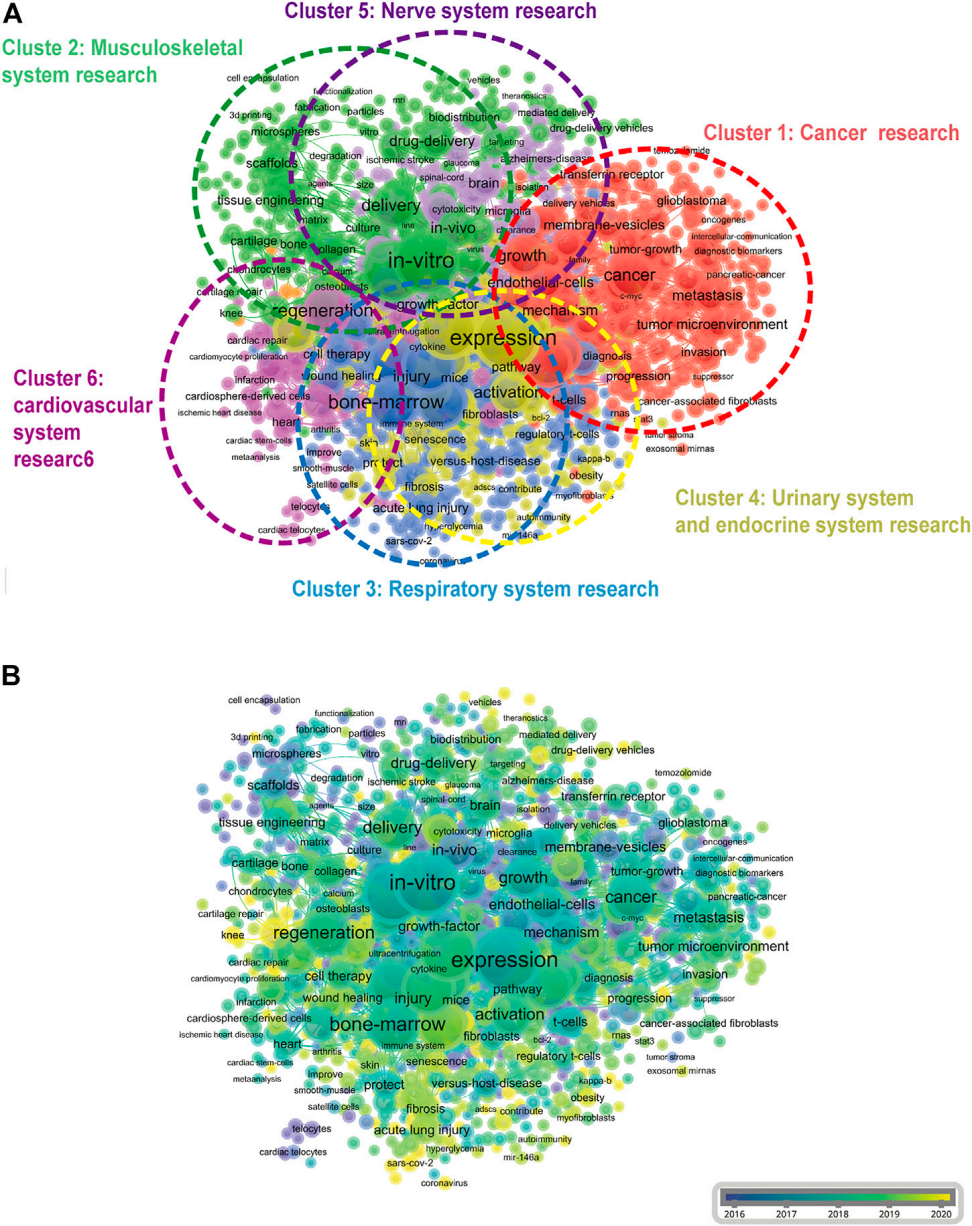
Co-occurrence analysis of global research about EVs based on stem cells. **(A)** Mapping of keywords in the research on EVs based on stem cells; the frequency is represented by point size and the keywords of research fields are divided into six clusters: cancer research (red), musculoskeletal system research (green), respiratory system research (dark blue), urinary system and endocrine system research (yellow), nerve system research (purple), and cardiovascular system research (pink) .**(B)** Distribution of keywords according to the mean frequency of appearance; keywords in yellow appeared later than those in blue.

The objective of co-occurrence analysis is to investigated popular directions and area of researches, and it also plays a vital role in monitoring the developments in scientific research. Keywords, which was defined as the words used more 10 times in titles/abstracts in all papers, were chosen and analyzed via VOS viewer. As shown in Figure 7A, the 1,255 identified keywords were mainly classified into six clusters as follows: cluster 1: cancer research (red), cluster 2: musculoskeletal system research (green), cluster 3: respiratory system research (dark blue), cluster 4: urinary system and endocrine system research (yellow), cluster 5: nerve system research (purple), and cluster 6: cardiovascular system research (pink). These results exhibited the most prominent research topics in EVs based on stem cells research so far. In the “cancer research” cluster, the primary keywords were: cancer, micrornas, and growth. For the “musculoskeletal system research” cluster, the frequently used keywords were: *in-vitro*, differentiation, and delivery. As for the “respiratory system research” cluster, the main used keywords were: therapy, bone-marrow, and inflammation. For the “urinary system and endocrine system research” cluster, the dominantly used keywords were: expression, angiogenesis and apoptosis. When talked about the “nerve system research” cluster, the frequently used keywords were: functional recovery, brain, and model. Furthermore, in the “cardiovascular system research” cluster, the primary used keywords were: regeneration, progenitor cells and myocardial-infarction. These results exhibited that the most prominent fields of EVs based on stem cells included the abovementioned six directions.

Additionally, keywords were coded with different colors by VOS viewer based on average times they appeared in all the published papers (Figure 7B). The color in blue meant that the keywords appeared earlier, whereas the color in yellow indicating later appearance. As shown in Figure 7B, the trends of most studies in the six clusters did not change dramatically, meaning that every research fields would be evenly concerned in the future**.**

**FIGURE 8:**
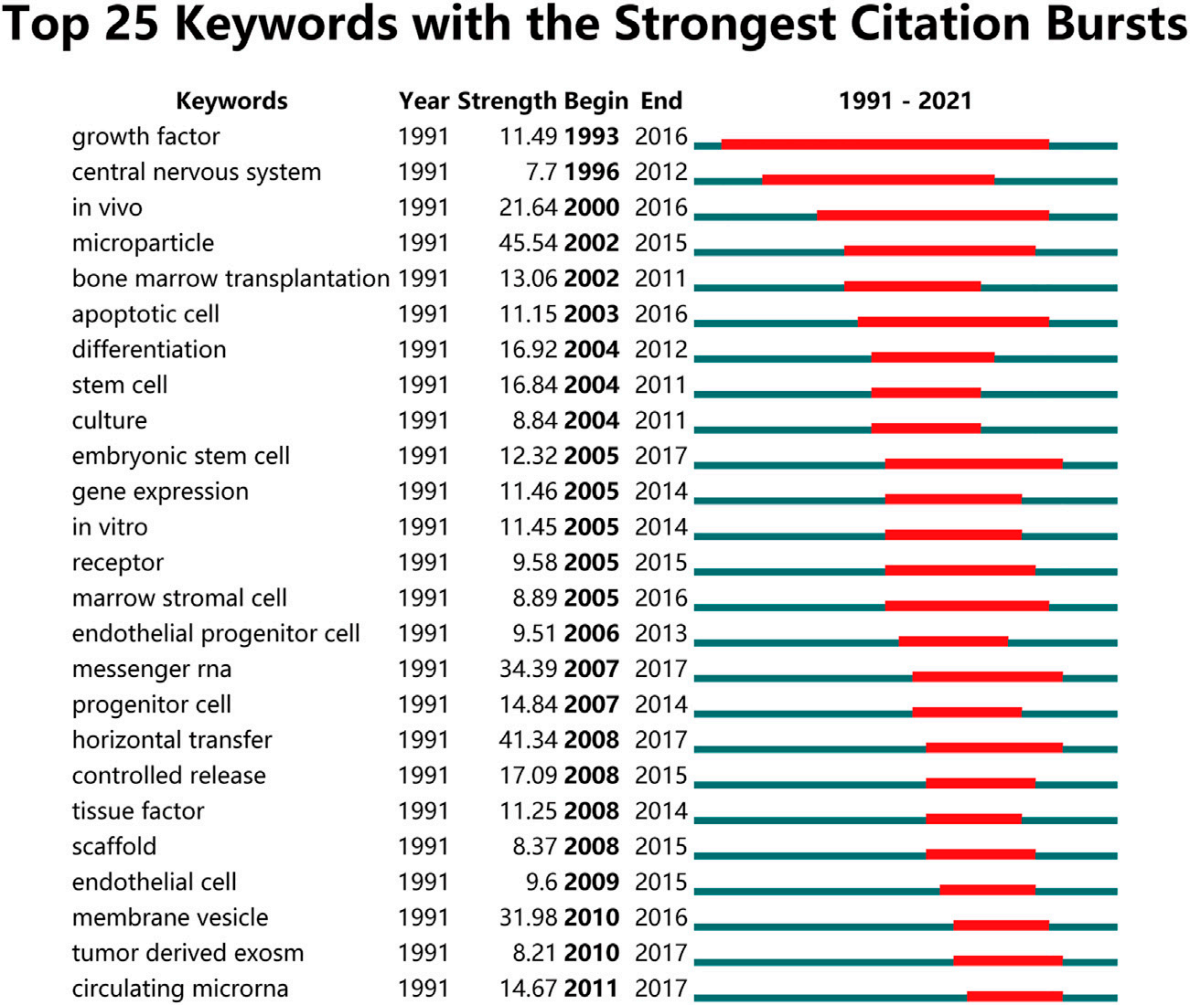
Citespace analysis of global research about EVs based on stem cells. Top 25 Keywords with the Strongest Citation Bursts.

We then detected the burst of keywords using CiteSpace’s algorithm of burst detection, and the minimum burst duration was 2 years. The top 25 keywords with strongest citation bursts was shown in Figure 8, where the blue lines represent the timeline and the red part in it represents the burst duration of the keyword. The most intense keyword was microparticle (strength = 45.54), followed by horizontal transfer (41.34) and messenger RNA (34.39). Additionally, the keyword with the longest burst time was growth factor, which lasted 23 years from 1993 to 2016 (Figure 8).

## Discussion

### Trend of global publications

Stem cells have been widely explored and achieved better therapeutic effects for many diseases in basic and clinical researches (Blau and Daley, 2019). However, there are some concerns associated with stem cells such as long-term culture *ex vivo*, increased immunogenicity of differentiated cells, as well as low therapeutic efficacy limited their application (Musiał-Wysocka et al., 2019). Therefore, EVs, including exosomes, microvesicles, microparticles, ectosomes, oncosomes and apoptotic bodies, have been proposed to replace conventional stem cell-based therapy for disease treatment due to the following advantages: fewer side effects and better safety, lower immunogenicity, protection of therapeutic agents, easier preservation methods, fewer ethical issues and ability to cross some biological barriers (Nguyen et al., 2021). In recent years, numerous researchers have been offered insightful publications in the field of EVs and stem cells (Mianehsaz et al., 2019). Wang et al. reported the global status and trends in exosomes field from 1994 to 2017 (Wang et al., 2019). As we mentioned before, EVs includes exosomes, microvesicles, etc. They mainly focused on the global trends study of exosomes field, which belongs to one of the EVs terms. Zhang et al. investigated the current research on mesenchymal stem cell-derived EVs (MSC-EVs) from 2009 to 2021 (Zhang et al., 2022). MSC-EVs showed a steep growth trend and the therapeutic effects and mechanisms were mainly focused on. They also predicted that the multidisciplinary integration and senescence-related topics might be mainly investigated in future. MSCs are a subtype of adult stem cells deriving from the mesoderm, with multi-directional differentiation and self-renewal ability (Li and Hua, 2017). However, the global development trends and visualized analysis of EVs based on stem cells (not only MSCs) have not been well studied yet. Therefore, the bibliometric and visualized analysis was applied in the present study to present the current status and make the predictions in future development in research of EVs based on stem cells. As shown in our study, a dramatic increase in number of publications per year has been observed. Furthermore, the RRIs have also increased significantly over the past few years. In this study, approximately 93 countries and 5,413 institutions were shown to have published papers in this field. In particular, China contributed the most papers (34.113%) than the United States (25.597%), Italy (8.614%), Germany (5.373%) and South Korea (4.362%). There is increasing evidence that the paracrine effects of stem cells are mediated by the secretion of EVs (Baglio et al., 2012). Intercellular communication has been observed in EVs under various physiological and pathological conditions. EVs from stem cells have been studied in various disease models (Chang et al., 2018). Above evidence could be inferred that more in-depth studies about EVs based on stem cells could be published in future and which in turn instruct researchers to conduct high-quality research.

### Quality and status of global publications

In order to figure out the quality and academic impact of different countries, the number of total citations and H-index were performed and the results shown in Figure 1 and Figure 2. Although China contributed the most publications, the USA possessed the largest total citations and H-index. Interestingly, in terms of average citations, Singapore ranked first, followed by Sweden, and Netherlands. Therefore, the USA might play the leading role of the world in the field of EVs based on stem cells, and China with the largest total number of publications will probably catch up with or even surpass the United States. In addition, we can clearly see that China, ranking second in terms of total citations and H-index, make a great progression in this field for it only ranked fourth and fifth, respectively, according to a reported in 2019 (Wang et al., 2019b). This contribution between the quantity and quality of publications from China might be due to the Chinese academic evaluation systems (CAESs) tend to focusing both in recent years (Synnestvedt et al., 2005). Even though China has achieved great progression, there is still a long way for China to improve the quality of studies in this field. With the reformation of CAESs and gradual increase in research funds in China, the quality of publications might be improved dramatically in this field (Xing et al., 2018).

We also investigated the journals associated with publications, and the results were shown in Figure 3. The journal of Stem Cell Research & Therapy, International Journal of Molecular Sciences, and Stem Cells International published more papers on EVs based on stem cells. Interestingly, the similar number of publications in top two journals has more than two times as many as the number of these published in the third-fifth ranked journals. The listed top 25 journals might be the most possible channels for future papers to be published.

The leading institutes from top five countries contributed greatly to the research on EVs based on stem cells, which was consistent with the global publications produced by top five countries. Approximately all top 25 institutes were coming from the top five countries, suggesting the pivotal role of first-class research institutes in improving one country’s academic ranking. Furthermore, the top ranked authors who contributed the most publications are Chinese and the top ranked institution of Shanghai Jiao Tong University together with the largest funds provided by National Natural Science Foundation of China (NSFC) suggested that China has played an important role in this research area. These authors listed in Figure 3A with the most publications in this field might be give prior attention to obtain the latest advancements in EVs based on stem cells.

The bibliometric research method could offer guidance for many research fields and it happens when two papers contain a common third articles of journal. Thus, it was performed to build up a similarity relationship among all publications in terms of institution, journal, country and author in present study. The data in Figure 4 demonstrated that International Journal of Molecular Sciences was the most related journal, Shanghai Jiao Tong University was the most related institution, China was the most related country, and Camussi, Giovanni was the most related author in this field. In addition, co-citation analysis was performed to investigate the impacts of publications through counting the total citation number. Present results in Figure 5 indicated that the landmark studies about EVs based on stem cells possessed the largest citation frequency in this field. Impressively, PLOS ONE might be the top journal with highest citation frequency. In terms of co-author analysis, it aimed to identify the collaboration between authors, institutions and countries. The authors/institutions/countries with higher total link strength tend to work collaboratively. Based on the results of the present study, we also provided views and suggestions for future researches, which may be important for the selection of research directions and the improvement of research level.

### Research focus on EVs based on stem cells

The directions and popular topics in EVs based on stem cells were discovered through the co-occurrence analysis. The map of an occurrence network was created based on the keywords in titles and abstracts of all included papers. From the results in Figure 7A and Figure 6 main research trends were observed as follows: cancer research, musculoskeletal system research, respiratory system research, urinary system and endocrine system research, nerve system research, and cardiovascular system research. These results not only complied with common sense in the field, but also clarify the directions of future investigation. It was obvious that within the center of co-occurrence map, which key words are predominant. Therefore, more and further high-quality research within these directions of this field are required. The overlay visualization map with great importance for monitoring the research directions was similar to that co-occurrence map except for the differently noted colors. As shown in Figure 7B, different colors indicated different scores and multi directions might be next topic in this topic. Recently, emergence of studies involving EVs and stem cells (including exosomes, exosomes, microvesicles, microparticles, ectosomes, oncosomes and apoptotic bodies) has been observed (Synnestvedt et al., 2005; Shao et al., 2018; Lin et al., 2021). EVs, containing a multitude of molecules, are natural micron-sized to nanoscale membrane vesicles which were encapsulated by phospholipid bilayers with the constitutive of inducible manner (Wu et al., 2021c). In addition, stem cells, one kind of most efficient producers of EVs, have been studied well in different cell types (Wang et al., 2017). EVs from embryonic mesenchymal stem cells alleviate osteoarthritis (Wang et al., 2017), mediate cartilage repair (Zhang et al., 2018), inhibit cell death (Tavakoli Dargani and Singla, 2019), enhance cardiac function after myocardial infarction (Khan et al., 2015), and promote pressure ulcer healing (Chen et al., 2019). EVs from induced pluripotent stem cells can improve myocardial infarction (Gao et al., 2020), accelerate skin cell proliferation (Kim et al., 2018), promote corneal epithelial hyperplasia (Tang et al., 2022), and resist osteoporosis (Cui et al., 2022). Similarly, EVs derived from MSCs have also been extensively studied. EVs from human umbilical cord mesenchymal stem cells enhance fracture healing through HIF-1α-mediated promotion of angiogenesis in a rat model of stabilized fracture (Zhang et al., 2019), EVs from bone marrow mesenchymal stem cell promote repair of damaged endometrium (Yao et al., 2019), promote metastasis of lung cancer cells (Zhang et al., 2019). EVs from adipose-derived mesenchymal stem cells ameliorate cardiac damage after myocardial infarction (Deng et al., 2019). EVs from TNF-α-treated human gingiva-derived MSCs inhibit periodontal bone loss (Nakao et al., 2021). As we showed above, “micrornas” and “delivery” of EVs on stem cells are still hotspots. The keyword burst detection showed the most intense keyword was microparticles, horizontal transfer and messenger RNA. In addition, Figure 8 also showed that circulating microRNA (miRNA) has been explosively cited since 2011, indicating that miRNA might also be an emerging research hotspot. MicroRNA played an important role in biological processes (such as cell proliferation, differentiation, apoptosis and so on) and contributed to the modulation of many key homeostatic processes through regulation of gene expression (Rahmani et al., 2020). For example, it could mediate age-related insulin resistance through high expression of miR-29–3p (Su et al., 2019), prevent group 2 innate lymphoid cell-dominant allergic airway inflammation via miR-146–5p (Fang et al., 2020), increase stomach cancer and reduce breast cancer respectively (Hanahan and Weinberg., 2000; Esquela-Kerscher and Slack., 2006; Pauli et al., 2011; Babashah et al., 2012). Therefore, miRNA related topics might be the future hotspots of EVs based on stem cells research. Numerous researches have been reported that EVs containing proteins, nucleic acids, lipids and metabolites might modulate the metabolic state of the body under different pathological conditions (Lo Cicero et al., 2015). We believe that further researches focusing on dissecting the components of EVs in different stem cells are urgently required, which could be helpful for investigating the therapeutic mechanism of EVs derived from stem cells on different tissues.

### Limitations of this study

Although this paper can provide unique insight into global trends and interests in EVs based on stem cells researches, there are still some limitations need to be mentioned. Firstly, it is quite difficult to distinguish the detailed information about EVs based stem cells, such as the origin of EVs and the relationship between them. Secondly, we might omit some publications due to the database and language bias. It is quite obvious that other major databases such as PubMed, Cochrane and Embase library as well as non-English language database have been excluded. Besides, some newly published high-quality papers might not be highlighted due to low citation frequency, which built difference between real-world research and bibliometric analysis. Therefore, it is quite necessary for researchers to address the latest publications and non-English papers in the future.

## Conclusion

In conclusion, our present study demonstrates increased global status and trends in EVs and stem cells researches from 1991 to 2021. China contributes the largest publications in the EVs based on stem cells research, and the United States with the highest total citation frequencies and H-index might also plays a vital role in this field. In addition, the journal Stem Cell Research Therapy has the most publications concerned about this issue. We can also predict the increasing trend of researches about EVs based on stem cells in the coming years. Notably, the research of EVs based on stem cells still gets global attention and future hotspots will be focused on many research directions including cancer research, musculoskeletal system research, respiratory system research, urinary system and endocrine system research, nerve system research, as well was cardiovascular system research. Furthermore, the keyword burst detection also showed that circulating miRNA might be an emerging research hotspot, and dissection of the components could be helpful for elucidating the therapeutic mechanism of EVs derived from stem cells.

## Data Availability

The raw data supporting the conclusion of this article will be made available by the authors, without undue reservation.
